# Early Postpartum Glucose Tolerance Reclassification by Gestational Diabetes Subtype

**DOI:** 10.1001/jamanetworkopen.2025.42668

**Published:** 2025-11-10

**Authors:** Julie A. D. Van, Joan C. Lo, Yeyi Zhu, Alexis S. King, Baiyang Sun, Emily Hashimoto-Roth, Hannes Rost, Stacey Alexeeff, Michael B. Wheeler, Erica P. Gunderson

**Affiliations:** 1 Department of Physiology, University of Toronto, Toronto, Ontario, Canada; 2 Metabolism Research Group, Division of Advanced Diagnostics, Toronto General Research Institute, Toronto, Ontario, Canada; 3 Division of Research, Kaiser Permanente Northern California, Pleasanton; 4 Department of Endocrinology, The Permanente Medical Group, Kaiser Permanente Oakland Medical Centre, Oakland, California; 5 Department of Health Systems Science, Kaiser Permanente Bernard J. Tyson School of Medicine, Pasadena, California; 6 Department of Molecular Genetics, University of Toronto, Toronto, Ontario, Canada

## Abstract

**Importance:**

Gestational diabetes (GD) is a heterogeneous condition that predisposes both mother and offspring to metabolic disorders. GD subtypes defined by antepartum testing results have been associated with adverse perinatal outcomes, but little is known about their relationship to maternal metabolic outcomes soon after pregnancy.

**Objective:**

To evaluate early postpartum glucose tolerance reclassification of GD subtypes.

**Design, Setting, and Participants:**

This cohort study examined women from the Study of Women, Infant Feeding, and Type 2 Diabetes Mellitus After GD Pregnancy (SWIFT), who were recruited within the Kaiser Permanente Northern California integrated health care system between 2008 and 2011. All women were diagnosed with GD using Carpenter and Coustan criteria with complete glucose measurements at all 4 time points of the diagnostic 3-hour 100-gram oral glucose tolerance test (OGTT). Data analyses were conducted from January to July 2025.

**Exposure:**

Three subtypes of GD based on the diagnostic OGTT: (1) postload glucose intolerance (GD-P), as having elevations only at 2 or more postload time points; (2) fasting hyperglycemia (GD-F), as having elevations at fasting and 1 postload time point; and (3) both (GD-M), as having elevations at fasting and 2 or more post-load time points.

**Main Outcomes and Measures:**

At 6 to 9 weeks after delivery, glucose tolerance classification was evaluated using 2-hour, 75-g OGTTs. Modified Poisson regression models were used to estimate adjusted prevalence ratios (PRs) of postpartum prediabetes associated with GD subtypes, without and with adjustments for age, race and ethnicity, prepregnancy body mass index, educational level, and gestational weight gain.

**Results:**

This study included 1005 women with GD (median [IQR] age, 33.2 [29.8-36.7] years; 368 [36.6%] Asian, 78 [7.8%] Black, 308 [30.6%] Hispanic, 16 [1.6%] multiracial, and 235 [23.4%] White). Prevalence of postpartum prediabetes was 34.5% (347 women), with wide variation across GD subtypes; 23.9% (147 of 616), 41.9% (52 of 124), and 55.8% (148 of 265) for GD-P, GD-F, and GD-M, respectively. Compared with women with GD-P, the adjusted PR for GD-F was 1.74 (95% CI, 1.36-2.24), and for GD-M, it was 2.23 (95% CI, 1.85-2.68) (both *P* < .001). Pairwise comparisons between GD-F and GD-M were also statistically significant (adjusted PR, 1.28; 95% CI, 1.01-1.61; *P* = .04).

**Conclusions and Relevance:**

In this cohort study, GD subtypes had distinct postpartum prediabetes risks. Early action and intervention to address dysglycemia may be most beneficial for women with fasting or mixed defects.

## Key Points

**Question:**

Do subtypes of gestational diabetes (GD) reclassify differently for glucose tolerance at 6 to 9 weeks after delivery?

**Findings:**

In this cohort study of 1005 women diagnosed with GD, 3 subtypes were defined using the diagnostic oral glucose testing results, as having isolated postload glucose intolerance (GD-P), fasting hyperglycemia (GD-F), or both defects (GD-M). These subtypes exhibited statistically significant graded increases in adjusted prevalence of postpartum prediabetes with GD-P as most favorable profile, GD-F as intermediate, and GD-M as most predisposed to prediabetes.

**Meaning:**

In this study, distinct postpartum prediabetes prevalence rates highlighted GD heterogeneity, calling for tailored early intervention and postpartum screening strategies by subtype.

## Teaser

This cohort study evaluates whether different subtypes of gestational diabetes are associated with different prevalence rates for postpartum prediabetes.

## Introduction

Gestational diabetes (GD) is a heterogeneous condition that predisposes both mother and child to adverse cardiometabolic health outcomes. Despite substantial efforts, predicting perinatal and long-term health outcomes has been challenging, as the risk factor profiles include various clinical, anthropometric, and socioeconomic traits.^1^ Subtyping GD at diagnosis may therefore provide a more personalized risk assessment and treatment regimen for future maternal care.

Despite nonuniversal definitions, 3 main subtypes of GD have emerged and generally reflect defects of fasting hyperglycemia, postload intolerance, or both during the diagnostic antepartum oral glucose tolerance test (OGTT). These subtypes have been mostly associated with distinct risks of pregnancy complications and peripartum outcomes, such as hypertensive disorders, cesarean delivery, preterm births, and large-for-gestational age infants.^2,3,4,5^ To date, only 1 study on GD subtypes^6^ has examined longer-term maternal outcomes up to 1 year after delivery. However, only 179 of the 613 pregnant women in that study had GD, and there were no significant differences in prediabetes and overt diabetes incidence at 3 months and 1 year among GD subtypes, likely owing to the small sample size.^6^ These studies on GD subtypes largely examined relatively homogeneous cohorts of women and exclusively defined subtypes based on the 2-hour, 75-g OGTT results. Therefore, the previously reported risk factors and outcomes of GD subtypes may not be fully actionable for clinical settings in the United States that serve diverse populations and diagnose GD using the 3-hour, 100-g OGTT.

Large and more diverse cohorts are therefore needed to better delineate the postpartum outcomes of GD subtypes, especially during the early postpartum period, to stratify risk for later diabetes onset. Both the American College of Obstetricians and Gynecologists and the American Diabetes Association recommend glucose tolerance retesting at 6 to 12 weeks after delivery after a GD delivery, yet less than 50% of women with GD follow through with this comprehensive test.^7,8^ Considering the current gaps in our understanding of GD subtypes and the opportunity for early intervention to prevent progression to diabetes, we therefore sought to extend GD subtyping definitions for women who are diagnosed with GD using the 3-hour, 100-g OGTT and to examine associations of GD subtypes with maternal glucose tolerance reclassification at 6 to 9 weeks after delivery.

## Methods

This cohort study was approved by the Kaiser Permanente Northern California Institutional Review Board and the Office of Research Ethics at University of Toronto, and all participants provided written informed consent. We followed the Strengthening the Reporting of Observational Studies in Epidemiology (STROBE) reporting guideline.

### Study Design

**Figure 1.**
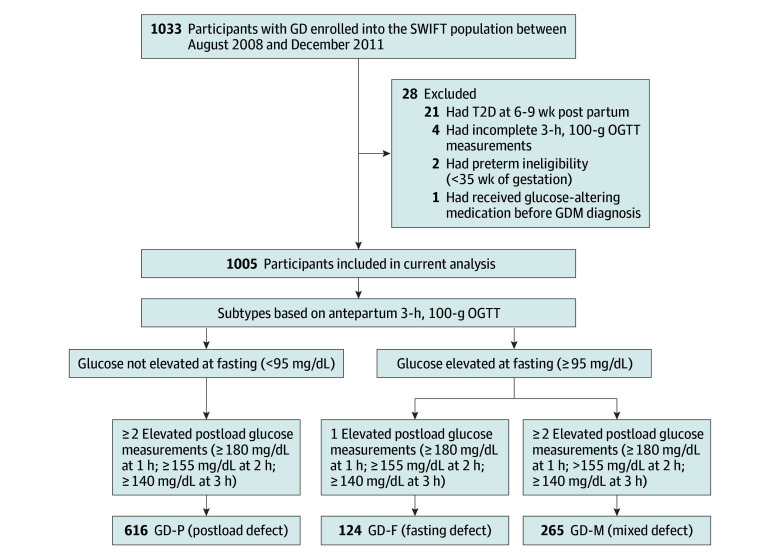
Study Flowchart GD indicates gestational diabetes; OGTT, oral glucose tolerance test; SWIFT, Study of Women, Infant Feeding, and Type 2 Diabetes after GD Pregnancy; T2D, type 2 diabetes.

All participants were recruited, screened for eligibility during pregnancy, and enrolled at 6 to 9 weeks after delivery to participate in the Study of Women, Infant feeding, and Type 2 Diabetes after GD Pregnancy (SWIFT) between August 2008 and December 2011. A study flow diagram is presented in Figure 1. The study evaluated maternal glucose tolerance reclassification at 6 to 9 weeks after delivery by GD subtype as an ad hoc analysis of SWIFT study participants. Full details on the SWIFT criteria are given in the eMethods in Supplement 1; the overall design, methods, participant characteristics, and outcomes have also been published elsewhere.^9,10,11^

### Study Population

Of the 1033 women who provided written informed consent for the SWIFT research study, 28 were excluded for having incomplete glucose measurements at all 4 time points of the diagnostic 3-hour, 100-g OGTT (n = 4); receiving glucose-altering medication prior to GD diagnosis (n = 1); delivering before 35 weeks of gestation (n = 2); and having type 2 diabetes at 6 to 9 weeks after delivery (diagnosed by 2-hour, 75-g research OGTT with repeat testing [n = 21]). The latter exclusion criterion avoided cases of undiagnosed diabetes before pregnancy. The final study sample included 1005 participants without early postpartum diabetes.

### GD Subtype Definitions

GD was diagnosed in accordance with Carpenter and Coustan criteria using the antepartum 3-hour, 100-g OGTT,^12^ with at least 2 elevated glucose measurements above or equal to 95 mg/dL at fasting and 180 mg/dL at 1-hour, 155 mg/dL at 2-hour, and/or 140 mg/dL at 3-hour postload time points (to convert glucose to mmol/L, multiply by 0.0055). Similar to previous studies, we defined GD subtypes based on the dominant metabolic defect of the diagnostic OGTT: (1) isolated postload glucose intolerance defects (GD-P) based on elevated measurements at least 2 postload time points only; (2) fasting hyperglycemia defects (GD-F) based on elevated measurements at fasting and any 1 postload time point; and (3) mixed defects (GD-M), with elevated measurements at fasting and at least two 2 postload measurements.

Additionally, we calculated homeostatic model assessment of insulin resistance (HOMA-IR) and homeostatic model assessment of β-cell function (HOMA-B) scores to evaluate insulin resistance and β-cell function using fasting glucose and insulin levels at 6 to 9 weeks after delivery. Higher HOMA-IR scores indicate higher insulin resistance, whereas higher HOMA-B scores indicate better function. The formulas are as follows: HOMA-IR = (fasting insulin × fasting plasma glucose)/405; and HOMA-B = 20 × (fasting insulin/[fasting plasma glucose – 63]).^13^

### Outcome Definitions

The primary outcome was prediabetes at 6 to 9 weeks after delivery after a GD pregnancy in accordance with American Diabetes Association criteria. Prediabetes was also further delineated as having impaired fasting glucose levels (IFG; between 100 and 125 mg/dL), impaired glucose tolerance (IGT; between 140 and 199 mg/dL), or both, ascertained by research 2-hour, 75-g OGTT. Isolated defects occur in the absence of other defects. For example, isolated IGT occurs with the absence of IFG (or with normal fasting glucose).

### Covariates

The SWIFT research staff administered surveys to collect sociodemographic (including race and ethnicity), pregnancy and delivery, socioeconomic, and lifestyle behavior data as well as obtained prenatal diagnosis and treatment of GD, pregnancy complications and delivery and newborn outcomes, medical history, and clinical data from the Kaiser Permanente Northern California electronic health records system. Biochemical assessments of fasting plasma samples were also carried out within several weeks of the research visit at 6 to 9 weeks after delivery (Northwest Lipid Research Laboratories). When assessing prevalence ratios (PRs), we selected risk factors associated with postpartum glucose intolerance such as maternal age, race and ethnicity (self-reported as Asian, Black, Hispanic, White, or multiracial [including Indigenous women]), prepregnancy body mass index (BMI; calculated as weight in kilograms divided by height in meters squared), education level, and gestational weight gain. Prepregnancy weight, height, and BMI were measured between 1 year prior to pregnancy and 14 weeks of gestation at Kaiser Permanente Northern California outpatient clinic visits. Postpartum anthropometric measurements were taken by trained SWIFT research staff at in-person visits at 6 to 9 weeks after delivery with participants wearing light clothing and at standing height without shoes. Waist circumference was measured in triplicate at the level of the right ischium on a bare abdomen using a Gulick II Plus 300 cm anthropometric tape (model 67019; Country Technology Inc). We categorized BMI using the classical cutoffs of the National Institutes of Health and World Health Organization, with obesity defined as 30 or greater.^14,15^ Gestational weight gain was also examined via survey and categorized in relation to BMI according to the 2009 Institute of Medicine Weight Gain Recommendations.^16^

### Statistical Analysis

We compared participant characteristics across the 3 GD subtypes using Kruskal-Wallis tests for continuous variables (as the vast majority were not normally distributed) and a χ^2^ test for categorical variables. Since the primary outcome is not rare, we estimated PRs of prediabetes at 6 to 9 weeks after delivery using modified Poisson regression models^17^ (which are more interpretable than odds ratios) for every pairwise comparison of subtypes. Unless otherwise specified, all adjusted PRs accounted for maternal age, race and ethnicity, prepregnancy BMI, educational level, and gestational weight gain. Effect modification by GD treatment (as a binary variable: medication-based treatment or diet only) was also assessed with the inclusion of the cross-product term for subtype × treatment in Poisson regression models. To address multiple comparisons and false discovery rate, we applied the Benjamini-Hochberg method and considered adjusted 2-sided *P* < .05 as statistically significant. All statistical analyses were performed using R, version 4.4.3 (R Project for Statistical Computing) using the rqlm and geepack packages. Data analyses were conducted from January to July 2025.

## Results

### GD Subtypes

This study included 1005 women with a median age of 33.2 (IQR, 29.8-36.7) years, 403 (40.1%) of whom had obesity before pregnancy. In terms of race and ethnicity, 368 women (36.6%) were Asian, 78 (7.8%) were Black, 308 (30.6%) were Hispanic, 235 (23.4%) were White, and 16 (1.6%) were multiracial. GD subtypes were defined by the dominant metabolic defect during the antepartum OGTT (eFigure 1 in Supplement 1): GD-P, 616 women (61.3%); GD-F, 124 women (12.3%); and GD-M, 265 women (26.4%). Comprehensive participant characteristics are found in Table 1 and eTables 1 to 3 in Supplement 1.

**Table 1.** Participant Characteristics

Characteristic	Participants, No. (%)				*P* value
	All (N = 1005)	By GD subtype			
		GD-P (n = 616)	GD-F (n = 124)	GD-M (n = 265)	
Age at delivery, median (IQR), y	33.2 (29.8-36.7)	32.9 (29.5-36.5)	33.1 (30.0-36.7)	33.7 (30.4-36.9)	.18
Race and ethnicity					
Asian	368 (36.6)	248 (40.3)	35 (28.2)	85 (32.1)	.005
Black	78 (7.8)	39 (6.3)	7 (5.6)	32 (12.1)	
Hispanic	308 (30.6)	177 (28.7)	41 (33.1)	90 (34.0)	
White	235 (23.4)	141 (22.9)	39 (31.4)	55 (20.7)	
Multiracial	16 (1.6)	11 (1.8)	2 (1.6)	3 (1.1)	
Prepregnancy BMI, median (IQR)	28.2 (24.4-33.4)	26.5 (23.7-31.1)	30.5 (25.8-37.2)	31.8 (27.0-36.2)	<.001
Obesity status	403 (40.1)	181 (29.4)	67 (54.0)	155 (58.5)	<.001
Attended or completed college	766 (76.2)	484 (78.6)	97 (78.2)	185 (69.8)	.01
Primiparous	366 (36.4)	239 (38.8)	37 (29.8)	90 (34.0)	.10
History of gestational diabetes, No. (%)^a^	160 (16.2)	102 (16.9)	16 (12.9)	42 (16.2)	.54
Gestational weight gain					
Below guidelines	318 (31.6)	206 (33.4)	38 (30.6)	74 (27.9)	.001
Within guidelines	347 (34.5)	232 (37.7)	36 (29.0)	79 (29.8)	
Above guidelines	340 (33.8)	178 (28.9)	50 (40.3)	112 (42.3)	
Received medication to treat GD	311 (30.9)	100 (16.2)	53 (42.7)	158 (59.6)	<.001

Abbreviations: BMI, body mass index (calculated as weight in kilograms divided by height in meters squared); GD, gestational diabetes; GD-F, GD with fasting hyperglycemia; GD-M, GD with mixed defects; GD-P, GD with isolated glucose intolerance.

^a^ GD history was missing for 20 women, including 14 with GD-P and 6 with GD-M.

eTable 1 in Supplement 1 summarizes the demographic and prenatal characteristics of each subtype. The prevalence of subtypes varied significantly by race and ethnicity (Table 1 and eFigure 2 in Supplement 1), with the starkest contrasts observed among Asian women (248 of 368 [67.4%], 35 of 368 [9.5%], and 85 of 368 [23.1%] with GD-P, GD-F, and GD-M, respectively) and Black women (39 of 78 [50.0%], 7 of 78 [9.0%], and 32 of 78 [41.0%] with GD-P, GD-F, and GD-M, respectively). Educational level differed across subtypes, as the GD-M subtype had lower percentages of women who attended or completed college than GD-P or GD-F (eTable 1 in Supplement 1). Women with the GD-P subtype had lower prepregnancy BMIs than those with GD-F and especially GD-M. Pairwise comparisons were significant across all combinations, underlining GD-F as an anthropometric intermediate. GD-P was the only subtype for which the majority of women did not have obesity (181 of 616 [29.4%] vs 67 of 124 [54.0%] for GD-F and 155 of 265 [58.5%] for GD-M). The 3 subtypes were comparable in terms of age at delivery, family history of diabetes, and neighborhood deprivation index (eTable 1 in Supplement 1).

eTable 2 in Supplement 1 summarizes the pregnancy characteristics of each subtype. Treatment for GD iterated differences between GD-P and other subtypes. We found that 516 women with the GD-P subtype (83.8%) managed their condition with a combination of diet and exercise, whereas pharmacologic treatments, such as oral medication and insulin, were heavily prescribed in the other 2 subtypes (53 [42.7%] for GD-F and 158 [59.7%] for GD-M). Despite differences in prepregnancy BMI, women had similar gestational weight gain across subtypes (eTable 2 in Supplement 1). However, the GD-P subtype had the lowest proportion of women who gained weight above guidelines (178 [28.9%] vs 50 [40.3%] for GD-F and 112 [42.3%] in GD-P; *P* = .001). Gestational age at GD diagnosis, parity, and length of pregnancy did not differ across subtypes (eTable 2 in Supplement 1).

eTable 3 in Supplement 1 summarizes maternal characteristics shortly after pregnancy. As part of the SWIFT procedures, we comprehensively measured anthropometric characteristics at 6 to 9 weeks after delivery and affirmed significant differences in weight, BMI, waist circumference, and body fat percentage (eTable 3 in Supplement 1). We found no significant differences in weight loss and retention, dietary factors, or breastfeeding exclusivity at 6 to 9 weeks after delivery across subtypes (eTable 3 in Supplement 1). Women with GD-P participated in less moderate-to-vigorous physical activity than women with other subtypes (median [IQR] metabolic equivalent hours per week, 20.6 [13.7-30.5] vs 24.9 [18.6-32.0] for GD-F and 23.4 [15.7-35.6] for GD-M; *P* = .003) (eTable 3 in Supplement 1).

### Peripartum Complications

eTable 4 in Supplement 1 shows the prevalence of pregnancy-related complications. Prevalence of cesarean delivery and postpartum depression were not significantly different among subtypes. Both GD-F and GD-M subtypes had higher rates of large-for-gestational age newborns (24 [19.4%] and 59 [22.3%], respectively) compared with the GD-P subtype (overall *P* < .001; GD-F vs GD-P: *P* = .01; GD-M vs GD-P: *P* < .001), but these rates were not significantly different from one another (*P* = .67). There were no other significant peripartum differences in the general health of neonates.

### Early Postpartum Maternal Dysmetabolism

Table 2 summarizes maternal metabolic outcomes shortly after pregnancy. Glucose and insulin levels were significantly different across subtypes at both fasting and 2-hour time points of the research 2-hour, 75-g OGTT. Fasting HOMA-IR scores followed a graded pattern (median [IQR], 3.9 [2.6-5.6] for GD-P; 5.0 [3.4-7.5] for GD-F; and 5.7 [4.0-9.2] for GD-M; *P* < .001), whereas fasting HOMA-B scores distinguished GD-M from the other subtypes (median [IQR], 223.3 [161.0-306.2] vs 222.3 [156.9-310.9] for GD-F and 245.0 [177.8-359.9] for GD-M; *P* = .005). Lipid biochemistry analyses revealed significant differences among subtypes for fasting high-density lipoprotein cholesterol and triglyceride levels, primarily between GD-P and the other 2 subtypes (Table 2). Adiponectin levels were distinctly lower in women with GD-M compared with those with the other subtypes (median [IQR], 6.5 [5.2-8.3] µg/mL vs 7.2 [5.6-9.0] µg/mL for GD-P and 7.2 [5.8-9.3] µg/mL for GD-F; *P* = .004), whereas leptin levels increased incrementally among the 3 subtypes (median [IQR], 22.6 [13.8-35.1] ng/mL for GD-P, 27.1 [17.0-41.0] ng/mL for GD-F, and 32.1 [22.4-42.2] ng/mL for GD-M; *P* < .001).

**Table 2.** Maternal Metabolic Profile of Fasting Plasma at 6 to 9 Weeks After Delivery^a^

Variable	Median (IQR)				*P *value
	All (N = 1005)	By GD subtype			
		GD-P (n = 616)	GD-F (n = 124)	GD-M (n = 265)	
Glycemic status					
Research 2-h, 75-g OGTT results					
Fasting glucose, mg/dL	94 (89-99)	92 (87-96)	98 (93-102)	98 (93-105)	<.001
2-h postload glucose, mg/dL	109 (93-128)	109 (92-127)	102 (92-114)	115 (97-139)	<.001
Fasting insulin, mIU/L	18.9 (13.2-29.0)	17.1 (11.8-24.5)	21.2 (14.4-30.1)	22.9 (17.2-36.1)	<.001
2-h Postload insulin, mIU/L	82.2 (54.4-123.7)	80.3 (52.4-118.9)	68.4 (47.1-92.2)	97.2 (64.3-139.6)	<.001
HOMA-IR score	4.4 (3.0-6.8)	3.9 (2.6-5.6)	5.0 (3.4-7.5)	5.7 (4.0-9.2)	<.001
HOMA-B score	227.3 (163.6-318.1)	223.3 (161.0-306.2)	222.3 (156.9-310.9)	245.0 (177.8-359.9)	.005
Lipid panel					
HDL cholesterol, mg/dL	51 (43-61)	54 (45-64)	49 (40-56)	48 (40-56)	<.001
LDL cholesterol, mg/dL	124 (104-146)	124 (104-146)	124 (104-146)	125 (106-146)	.87
Total cholesterol, mg/dL	201 (178-225)	200 (179-223)	201 (177-226)	203 (179-230)	.82
Triglycerides, mg/dL	99 (69-149)	92 (65-139)	109 (75-165)	112 (79-172)	<.001
Adiponectin, µg/mL	7.0 (5.5-8.8)	7.2 (5.6-9.0)	7.2 (5.8-9.3)	6.5 (5.2-8.3)	.004
Leptin, ng/mL	25.7 (15.8-38.0)	22.6 (13.8-35.1)	27.1 (17.0-41.0)	32.1 (22.4-42.2)	<.001

Abbreviations: GD, gestational diabetes; GD-F, GD with fasting hyperglycemia; GD-M, GD with mixed defects; GD-P, GD with isolated glucose intolerance; HDL, high-density lipoprotein; HOMA-B, homeostatic model assessment of β cell function; HOMA-IR, homeostatic model assessment of insulin resistance; LDL, low-density lipoprotein; OGTT, oral glucose tolerance test.

SI conversation factors: To convert glucose to mmol/L, multiply by 0.0055; insulin to pmol/L, multiply by 6.945; HDL, LDL, and total cholesterol to mmol/L, multiply by 0.0259; and triglycerides to mmol/L, multiply by 0.0113.

^a^ There are missing values across all lipid panel variables (n = 8) as well as fasting (n = 6) and postload (n = 2) insulin levels.

### Associations With Prediabetes

**Figure 2.**
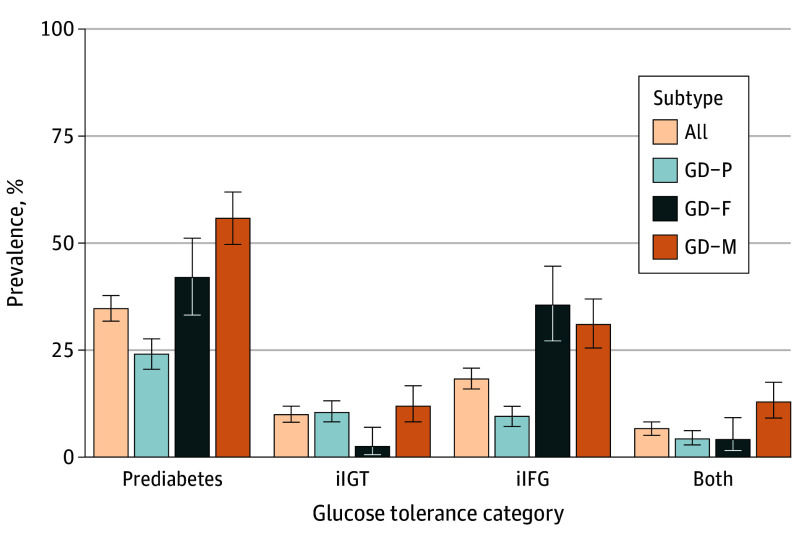
Prevalence of Prediabetes at 6 to 9 Weeks After Delivery Prediabetes was further classified as having isolated impaired glucose tolerance (iIGT), isolated impaired fasting glucose (iIFG), and both. Error bars indicate 95% CIs.

Figure 2 shows the prevalence of overall prediabetes and specific glucose tolerance categories. The prevalence of prediabetes at 6 to 9 weeks after delivery was 34.5% overall (347 of 1005 women) and varied widely across GD subtypes (23.9% [147 of 616], 41.9% [52 of 124], and 55.8% [148 of 265] for GD-P, GD-F, and GD-M, respectively; *P* < .001). Compared with women with the GD-P subtype, women with the GD-F (adjusted PR, 1.74 [95% CI, 1.35-2.24]; *P* < .001) and GD-M (adjusted PR, 2.23 [95% CI, 1.86-2.69]; *P* < .001) subtypes had an increased risk of prediabetes at 6 to 9 weeks after delivery (Table 3). Pairwise comparisons between GD-F and GD-M were also statistically significant (adjusted PR, 1.28 [95% CI, 1.02-1.62]; *P* = .04).

**Table 3.** PRs Estimated Using Poisson Regression for Prediabetes at 6 to 9 Weeks After Delivery

Pairwise comparison^a^	Model 1		Model 2		Model 3		Model 4	
	PR (95% CI)	*P *value	PR (95% CI)	*P *value	PR (95% CI)	*P *value	PR (95% CI)	*P *value
GD-F vs GD-P	1.76 (1.37-2.26)	<.001	1.84 (1.43-2.35)	<.001	1.76 (1.37-2.26)	<.001	1.74 (1.35-2.24)	<.001
GD-M vs GD-P	2.34 (1.96-2.79)	<.001	2.39 (2.00-2.85)	<.001	2.28 (1.90-2.74)	<.001	2.23 (1.86-2.69)	<.001
GD-M vs GD-F	1.33 (1.05-1.68)	.02	1.30 (1.03-1.64)	.03	1.30 (1.03-1.64)	0.03	1.28 (1.02-1.62)	.04

Abbreviations: GD-F, gestational diabetes with fasting hyperglycemia; GD-M, gestational diabetes with mixed defects; GD-P, gestational diabetes with isolated glucose intolerance; PR, prevalence ratio.

^a^ Model 1: unadjusted. Model 2: adjusted for age, race and ethnicity. Model 3: model 2 plus prepregnancy body mass index. Model 4: model 3 plus educational level (high school or less vs some college or more) plus gestational weight gain categories (above/within/below guidelines). All PRs remained statistically significant after multiple comparison by the Benjamini-Hochberg method (adjusted *P* < .05).

Like GD, prediabetes manifests in different forms. In the current study, 9.9% (98 women), 18.2% (183 women), and 6.5% (65 women) were classified with isolated IGT, isolated IFG, or both, respectively, at 6 to 9 weeks after delivery (Figure 2). The GD-P and GD-M subtypes had higher percentages of postpartum isolated IGT (10.4% [64 women] and 12.1% [32 women], respectively) compared with the GD-F subtype (2.4% [3 women]) (Figure 2). Postpartum isolated IFG was observed in 9.3% (57 women), 35.5% (44 women), and 30.9% (124 women) of participants with the GD-P, GD-F, and GD-M subtypes, respectively (Figure 2). Having both IFG and IGT was most common in women with GD-M (12.8% [34 women]) compared with those with single defects (GD-P, 4.2% [26 women]; GD-F, 4.0% [5 women]) (Figure 2).

Given that the subtypes had different treatment profiles for GD, we also evaluated the influence of GD treatment type on the associations between subtypes and postpartum prediabetes. eTable 5 in Supplement 1 shows ratios among subtypes when adjusted for GD treatment type, with no significant effect modification between GD subtypes and prevalence of postpartum prediabetes.

## Discussion

To our knowledge, this study is the first to categorize women diagnosed with GD according to the 3-hour, 100-g OGTT and characterize their sociodemographic, pregnancy, and early postpartum traits by subtype. We first identified significant subtype differences in race and ethnicity and anthropometry, underscoring 2 key determinants for specific defects during pregnancy. Additionally, these pregnancy defects did not fully resolve after delivery. The 3 GD subtypes exhibited graded increases in prediabetes prevalence at 6 to 9 weeks after delivery, with GD-P as the most favorable subtype and GD-M as the most predisposed subtype. These findings offer insights for improved risk stratification during the early postpartum period to prioritize longer-term monitoring and early intervention in specific groups of women with GD, with the goal of preventing progression to diabetes after GD pregnancy.

Previous studies on GD subtypes predominantly investigated cohorts of pregnant women with GD diagnosed using the 2-hour, 75-g OGTT, reflecting the standard practices of Canada,^2,6,18^ China,^3^ Mexico,^19^ Germany and Austria,^20^ and Belgium.^21^ These independent cohorts were also homogeneous, as more than 70% of participants belonged to a majority racial and ethnic group. We not only characterized a racially and ethnically diverse cohort that represents the US population but also found significant differences in subtype distribution across races and ethnicities. Our findings align with a retrospective cohort study of more than 11 000 pregnant women with various glycemic subtypes, whereby Asian women had the highest prevalence of all abnormal glucose tolerance phenotypes, except for GD characterized by fasting hyperglycemia.^22^ In the current study, fasting hyperglycemia was less prevalent in Asian women, as nearly two-thirds of women in this demographic had the GD-P subtype. Notably, GD-P stood alone as the only subtype for which the majority of women did not have obesity. These findings highlight the associations between race and ethnicity and obesity as important determinants of GD subtypes.

Borrowing from our understanding of glucose tolerance categories for prediabetes, GD subtypes may also have distinct etiologies. Postload glucose intolerance predominantly reflects peripheral insulin resistance, whereby postprandial glucose levels remain elevated because of impaired glucose uptake by muscles.^23,24^ Reduced insulin sensitivity has indeed been associated with lower muscle mass.^25,26^ Although we did not directly measure muscle mass, women with GD-P participated in significantly less moderate-to-vigorous physical activity and had smaller waist circumferences than those with other subtypes. On the other hand, fasting hyperglycemia generally reflects hepatic insulin resistance, whereby the liver overproduces glucose because of insufficient suppression.^27^ Chronic inflammation stemming from visceral obesity is believed to be a top culprit for dysfunctional insulin signaling in the liver,^24^ which aligns with the higher adiposity observed in the GD-F and GD-M subtypes. Further work is warranted to assess and compare the inflammatory profiles across GD subtypes, especially between those with and without antepartum fasting hyperglycemia.

GD subtypes conferred distinct maternal glucose tolerance outcomes. Furthermore, the main pregnancy defects that defined each subtype persisted after delivery at different ratios among subtypes, which, depending on the subtype, suggests an incomplete return to normoglycemia or underlying dysmetabolism from before pregnancy. Studies have shown that skeletal muscle insulin resistance may be one of the first, if not initiating, events, supposedly preceding β-cell failure and overt hyperglycemia by decades.^28,29^ Metabolically stressful events such as pregnancy may uncover or amplify these subclinical defects of peripheral insulin resistance as indicated by postload glucose intolerance during an OGTT. The reversal of such defects may occur naturally after delivery in the GD-P subtype, with greater success when implementing lifestyle changes that increase muscle mass and peripheral sensitivity.^30^

Meanwhile, the other 2 subtypes may have a more chronic disposition. Women with GD-F and especially GD-M had higher percentages of obesity and excessive weight gain during pregnancy as well as lower high-density lipoprotein cholesterol, higher triglyceride, and higher leptin levels at 6 to 9 weeks after delivery compared with women with GD-P. Delivery alone may therefore only minimize but not completely remove the long-standing dysmetabolic state of these subtypes. Of note, GD-F and GD-P had similar levels of adiponectin at 6 to 9 weeks after delivery, suggesting some degree of preserved β-cell function and overall metabolic health that is lacking in GD-M.^31,32,33^ We also observed that GD-F and GD-M had similar prevalence of postpartum prediabetes after adjusting for GD treatment. This finding underscores the importance of early pharmacologic intervention as soon as GD is diagnosed. These subtypes may achieve improved glycemic control by combining lifestyle and pharmacologic approaches that improve insulin sensitivity and mitigate obesity.

### Limitations

The study has some limitations. First, although our study is the first (to our knowledge) to subtype GD using the 3-hour, 100-g OGTT and to demonstrate significant differences in prediabetes risks, our findings may nonetheless have reduced generalizability for clinical settings that diagnose GD using the 2-hour, 75-g OGTT; therefore, we encourage further characterizations and comparisons of women diagnosed with GD by different criteria. Second, although we examined a racially and ethnically diverse cohort, the relatively small sample sizes of Black and Indigenous women invite a more focused analysis in the future. Third, insulin measurements were not conducted during antepartum testing (as these are not standard procedures in clinical settings); therefore, we could not directly investigate the underlying mechanisms of glycemic dysregulation during pregnancy.

## Conclusions

In this cohort study of 1005 women, we demonstrated that GD subtypes were associated with distinct risks of prediabetes at 6 to 9 weeks after delivery. More specifically, women with impaired fasting glucose levels at GD diagnosis (subtypes GD-F and GD-M) had higher risks of early postpartum prediabetes than those with isolated glucose intolerance (GD-P subtype). These findings highlight specific groups of pregnant women with fasting defects who would most benefit from immediate lifestyle interventions and early postpartum testing.

## Notes

Supplement 1. **eFigure 1. **Oral Glucose Tolerance Test Curves by GD Subtype **eFigure 2.** Prevalence of GD Subtypes by Race and Ethnicity **eTable 1. **Prepregnancy, Medical History, and Sociodemographic Characteristics **eTable 2.** Pregnancy Characteristics **eTable 3.** Early Postpartum Characteristics **eTable 4. **Prevalence of Maternal and Newborn Health Outcomes **eTable 5. **Prevalence Ratios Adjusting for Effect Modification of GD Treatment Estimated Using Poisson Regression for Prediabetes at 6 to 9 Weeks Postpartum
eMethods

Supplement 2.
Data Sharing Statement
