# Supplementary material for: Early Postpartum Glucose Tolerance Reclassification by Gestational Diabetes Subtype
Source: JAMA Netw Open. 2025 Nov 10;8(11):e2542668. doi: 10.1001/jamanetworkopen.2025.42668 (PMC12603860; doi:10.1001/jamanetworkopen.2025.42668)
Supplement: Supplement 1. — eFigure 1. Oral Glucose Tolerance Test Curves by GD Subtype eFigure 2. Prevalence of GD Subtypes by Race and Ethnicity eTable 1. Prepregnancy, Medical History, and Sociodemographic Characteristics eTable 2. Pregnancy Characteristics eTable 3. Early Postpartum Characteristics eTable 4. Prevalence of Maternal and Newborn Health Outcomes eTable 5. Prevalence Ratios Adjusting for Effect Modification of GD Treatment Estimated Using Poisson Regression for Prediabetes at 6 to 9 Weeks Postpartum eMethods [file jamanetwopen-e2542668-s001.pdf]

Supplemental Online Content

Van JAD, Lo JC, Zhu Y, et al. Early postpartum glucose tolerance reclassification by gestational diabetes subtype. *JAMA Netw Open*. 2025;8(11):e2542668. doi:10.1001/jamanetworkopen.2025.42668

eFigure 1. Oral Glucose Tolerance Test Curves by GD Subtype

eFigure 2. Prevalence of GD Subtypes by Race and Ethnicity

eTable 1. Prepregnancy, Medical History, and Sociodemographic Characteristics

eTable 2. Pregnancy Characteristics

eTable 3. Early Postpartum Characteristics

eTable 4. Prevalence of Maternal and Newborn Health Outcomes

eTable 5. Prevalence Ratios Adjusting for Effect Modification of GD Treatment Estimated Using Poisson Regression for Prediabetes at 6 to 9 Weeks Postpartum

eMethods

This supplemental material has been provided by the authors to give readers additional information about their work.

© 2025 Van JAD et al. *JAMA Network Open*.

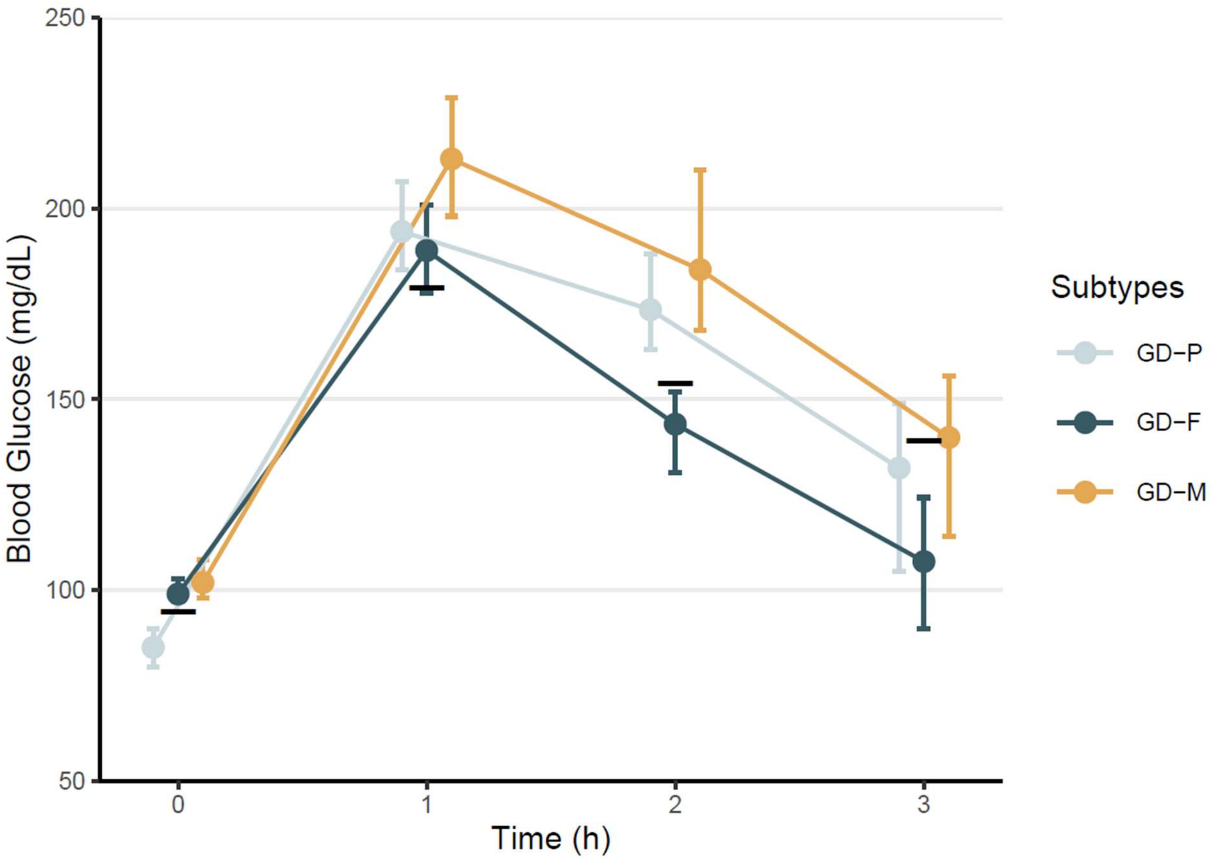

eFigure 1. Oral Glucose Tolerance Test Curves by GD Subtype. Median values and interquartile ranges are shown for each subtype. Black flat lines indicate the thresholds for each timepoint of the 3-hour 100-gram oral glucose tolerance test based on Carpenter and Coustan criteria.

© 2025 Van JAD et al. *JAMA Network Open*.

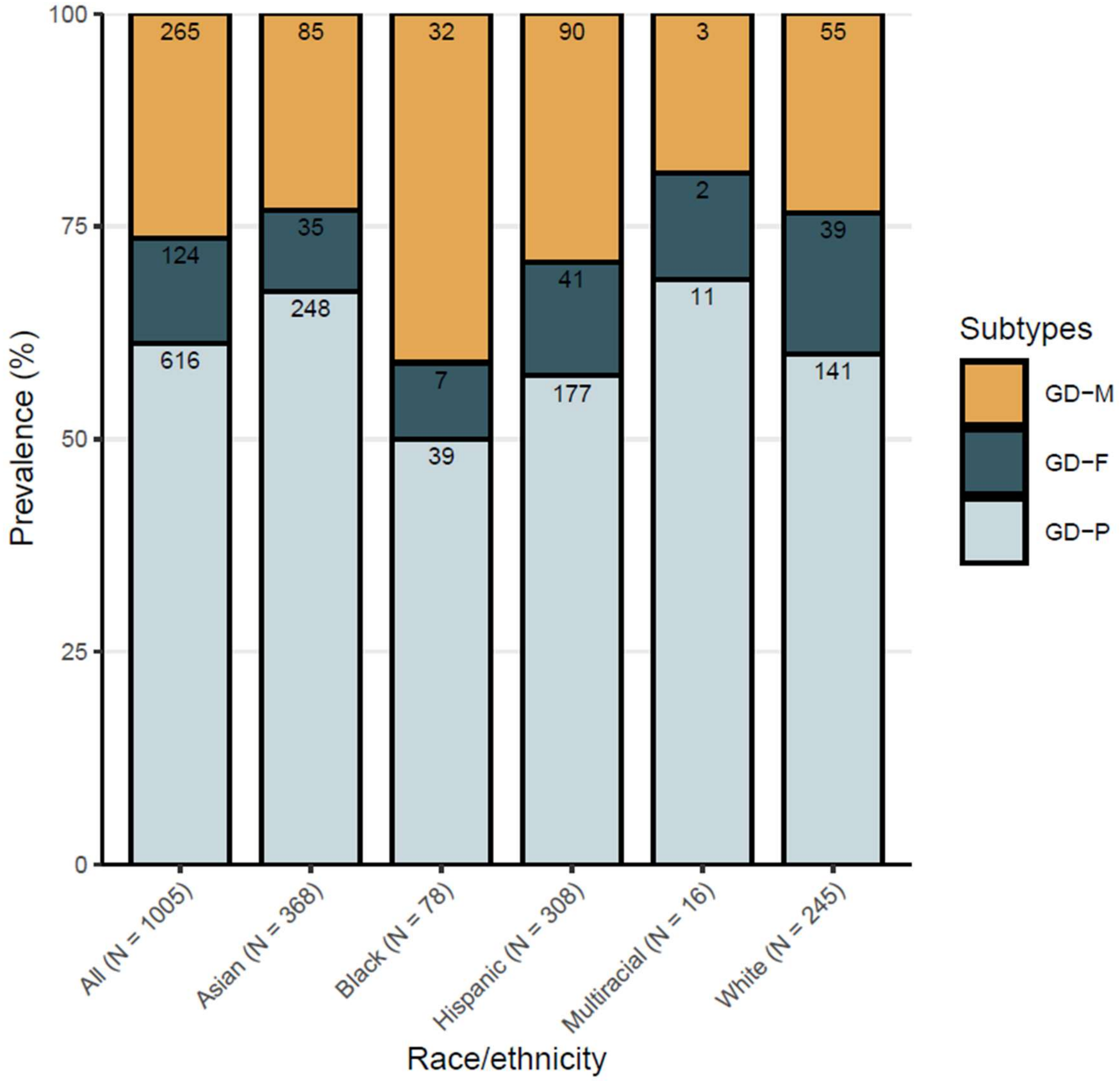

eFigure 2. Prevalence of GD Subtypes by Race and Ethnicity

Absolute numbers are shown for each category.

© 2025 Van JAD et al. *JAMA Network Open*.

eTable 1. Prepregnancy, Medical History, and Sociodemographic Characteristics

Variables	All (n = 1005)	GD-P (n = 616)	GD-F (n = 124)	GD-M (n = 265)	P
**Physical traits**					
Age at delivery, median (IQR), y	33.2 (29.8, 36.7)	32.9 (29.5, 36.5)	33.1 (30.0, 36.7)	33.7 (30.4, 36.9)	0.18
Race/Ethnicity, No. (%)					0.005
Asian	368 (36.6)	248 (40.3)	35 (28.2)	85 (32.1)	
Black	78 (7.8)	39 (6.3)	7 (5.6)	32 (12.1)	
Hispanic	308 (30.6)	177 (28.7)	41 (33.1)	90 (34.0)	
Multiracial	16 (1.6)	11 (1.8)	2 (1.6)	3 (1.1)	
White	235 (23.4)	141 (22.9)	39 (31.5)	55 (20.7)	
Pre-pregnancy BMI, median (IQR), kg/m^2	28.2 (24.4, 33.4)	26.5 (23.7, 31.1)	30.5 (25.8, 37.2)	31.8 (27.0, 36.2)	<.001
Obesity status, No. (%)	403 (40.1)	181 (29.4)	67 (54.0)	155 (58.5)	<.001
**Socioeconomic status**					
Married or living with partner, No. (%)*	917 (91.4)	568 (92.4)	110 (89.4)	239 (90.2)	0.40
Attended or completed college, No. (%)	766 (76.2)	484 (78.6)	97 (78.2)	185 (69.8)	0.01
Neighborhood Deprivation Index, No. (%)					0.07
Lowest deprivation (≤ - 1.0)	108 (10.7)	73 (11.9)	10 (8.1)	25 (9.4)	
Below average deprivation (- 1.0 to 0)	458 (45.6)	283 (45.9)	67 (54.0)	108 (40.8)	
Above average deprivation (0 to 1.0)	293 (29.2)	169 (27.4)	37 (29.8)	87 (32.8)	
Highest deprivation (> 2.0)	146 (14.5)	91 (14.8)	10 (8.1)	45 (17.0)	
WIC program recipient, No. (%)	259 (25.8)	151 (24.5)	29 (23.4)	79 (29.8)	0.21
**Medical History**					
Family history of diabetes, No. (%)	494 (49.2)	302 (49.0)	54 (43.5)	138 (52.1)	0.29
History of polycystic ovarian syndrome, No. (%)	66 (6.6)	36 (5.8)	8 (6.5)	22 (8.3)	0.40
History of gestational diabetes, No. (%)*	160 (16.2)	102 (16.9)	16 (12.9)	42 (16.2)	0.54

*There are missing values for marital status (n = 2) and history of gestational diabetes (n = 20).

© 2025 Van JAD et al. *JAMA Network Open.*

© 2025 Van JAD et al. *JAMA Network Open*.

eTable 2. Pregnancy Characteristics

Variables	All (n = 1005)	GD-P (n = 616)	GD-F (n = 124)	GD-M (n = 265)	P
**Clinical**					
Primiparous, No. (%)	366 (36.4)	239 (38.8)	37 (29.8)	90 (34.0)	0.10
Length of pregnancy, median (IQR)	39.1 (38.6, 39.9)	39.1 (38.4, 40.0)	39.3 (39.0, 39.9)	39.1 (38.4, 39.7)	0.18
**Anthropometry**					
Gestational weight gain, median (IQR), kg	10.0 (6.4, 13.6)	10.0 (6.8, 13.6)	9.5 (5.0, 13.8)	10.0 (5.9, 14.5)	0.68
Gestational weight gain, No. (%)					0.001
Below guidelines	318 (31.6)	206 (33.4)	38 (30.6)	74 (27.9)	
Within guidelines	347 (34.5)	232 (37.7)	36 (29.0)	79 (29.8)	
Above guidelines	340 (33.8)	178 (28.9)	50 (40.3)	112 (42.3)	
**GD Diagnosis and Treatment Variables**					
Gestational age at diagnosis, median (IQR), weeks	27.9 (25.3, 29.4)	27.9 (25.6, 29.4)	28.1 (25.3, 29.6)	27.6 (24.1, 29.0)	0.23
Antepartum 3-hour 100-gram oral glucose tolerance test (OGTT), median (IQR)					
Fasting glucose, mg/dL	92 (83, 99)	85 (80, 90)	99 (97, 103)	102 (98, 108)	<.001
1-hour post-load glucose, mg/dL	197 (185, 213)	194 (184, 207)	189 (178, 201)	213 (198, 229)	<.001
2-hour post-load glucose, mg/dL	173 (161, 190)	173 (163, 188)	144 (163, 188)	184 (168, 210)	<.001
3-hour post-load glucose, mg/dL	130 (103, 149)	132 (105, 149)	108 (90, 124)	140 (114, 156)	<.001
3-hour 100-gram OGTT z-score sum, median (IQR)	-0.5 (-1.7, 1.2)	-1.0 (-1.9, 0.2)	-1.7 (-2.4, -0.8)	1.9 (0.6, 4.1)	<.001
Received medication to treat GD, No. (%)	311 (30.9)	100 (16.2)	53 (42.7)	158 (59.2)	<.001

© 2025 Van JAD et al. *JAMA Network Open*.

eTable 3. Early Postpartum Characteristics

Variables	All (n = 1005)	GD-P (n = 616)	GD-F (n = 124)	GD-M (n = 265)	P
**Anthropometry**					
Weight, median (IQR), kg	72.5 (63.4, 86.6)	69.5 (61.0, 80.1)	79.8 (70.5, 95.1)	80.5 (69.7, 96.3)	<.001
Height, median (IQR), cm	159 (155, 164)	159 (154, 163)	160 (157, 165)	160 (156, 165)	0.009
BMI, median (IQR), kg/m^2	28.8 (25.4, 33.9)	27.6 (24.4, 31.2)	30.2 (27.2, 36.7)	32.0 (27.7, 35.9)	<.001
Weight retention pre-pregnancy to study baseline (6-9 weeks), median (IQR), kg	1.4 (-2.3, 4.8)	1.6 (-1.9, 4.8)	1.2 (-4.4, 4.7)	0.7 (-3.2, 5.0)	0.28
Weight loss post-delivery, median (IQR), kg	9.0 (6.8, 11.4)	8.9 (7.0, 10.8)	9.2 (7.0, 11.7)	9.3 (6.5, 12.1)	0.30
Waist circumference, median (IQR), cm*	87.5 (79.7, 97.1)	84.8 (78.2, 93.0)	90.9 (84.2, 101.1)	93.8 (83.8, 103.2)	<.001
Body fat, median (IQR), %*	45.5 (40.9, 50.6)	44.4 (39.8, 48.8)	45.7 (41.7, 53.4)	48.6 (43.7, 52.7)	<.001
**Lifestyle and behavioural activity**					
Total fat intake, median (IQR), % kcal*	26.8 (21.1, 32.9)	26.4 (20.4, 32.6)	26.4 (21.8, 33.3)	27.7 (21.8, 33.7)	0.11
Dietary fiber intake, median (IQR), g/100 kcal*	0.99 (0.76, 1.23)	0.98 (0.78, 1.23)	1.00 (0.75, 1.27)	1.00 (0.75, 1.21)	0.95
Dietary glycemic index, median (IQR) *	227.6 (170.2, 300.3)	229.8 (170.6, 309.1)	238.3 (184.6, 297.2)	215.3 (165.0, 279.1)	0.06
Moderate-to-vigorous physical activity, median * (IQR), metabolic equivalent h/wk	22.2 (14.5, 32.0)	20.6 (13.7, 30.5)	24.9 (18.6, 32.0)	23.4 (15.7, 35.6)	0.003
Current or past smoking history, No. (%)*	201 (20.0)	112 (18.2)	29 (23.4)	60 (22.6)	0.19
Exclusively breastfeeding, No. (%)	215 (21.4)	143 (23.2)	22 (17.7)	50 (18.9)	0.33

There are missing values for waist circumference (n = 7), body fat (n = 154), dietary characteristics (such as glycemic index, animal fat intake, and fiber intake: n = 7), and physical activity (n = 6).

© 2025 Van JAD et al. *JAMA Network Open*.

eTable 4. Prevalence of Maternal and Newborn Health Outcomes

Perinatal outcomes, No. (%)*	All (N = 1005)	GD-P (N = 616)	GD-F (N = 124)	GD-M (N = 265)	P
**Maternal health**					
C-section delivery	321 (31.9)	181 (29.4)	41 (33.1)	99 (37.4)	0.06
Prolonged length of stay^	480 (47.8)	276 (44.8)	6 (45.2)	148 (55.8)	0.009
Postpartum depression	133 (13.7)	79 (13.2)	20 (16.9)	34 (13.4)	0.54
**Newborn Health**					
NICU stay	49 (4.9)	26 (4.2)	8 (6.5)	15 (5.7)	0.45
Prolonged length of stay^	512 (50.9)	296 (48.1)	60 (48.4)	156 (58.6)	0.01
Neonatal hypoglycemia	191 (19.1)	106 (17.2)	22 (17.7)	63 (23.8)	0.07
Large for gestational age	149 (14.8)	66 (10.7)	24 (19.4)	59 (22.3)	<.001

^Prolonged length of stay for mothers was defined based on delivery method: vaginal (at least two days) and c-section (at least four days). Prolonged length of stay for newborns was defined as two or more days.

*There are missing or unknown values in postpartum depression (n = 33) and neonatal hypoglycemia (n = 1).

© 2025 Van JAD et al. *JAMA Network Open*.

eTable 5. Prevalence Ratios Adjusting for Effect Modification of GD Treatment Estimated Using Poisson Regression for Prediabetes at 6 to 9 Weeks Postpartum

Pairwise Comparison*	Model 5: PR (95% CI)	P	Model 6: PR (95% CI)	P	P for interaction
GD-F vs. GD-P	1.52 (1.18 – 1.97)	0.001^	1.63 (1.13 – 2.35)	0.009^	0.34
GD-M vs. GD-P	1.83 (1.50 – 2.28)	<.001^	2.12 (1.61 – 2.78)	<.001^	
GD-M vs. GD-F	1.23 (0.98 – 1.55)	0.08	1.30 (0.88 – 1.92)	0.19	

*Model 5: Adjusted for GD treatment (medication-based treatment or diet only). Model 6: Adjusted for the interaction of GD subtype * GD treatment. Overall p for interaction compares Models 5 and 6.

^All remained statistically significant after multiple comparisons by Benjamini-Hochberg method (adjusted p < 0.05).

© 2025 Van JAD et al. *JAMA Network Open*.

eMethods

SWIFT Inclusion Criteria

All SWIFT participants were Kaiser Permanente (KP) health plan members who delivered at KP Northern California (KPNC) hospitals. More specifically, women were recruited during pregnancy based on electronic medical record data for GD diagnosis in real time and were subsequently contacted by research staff to screen for study eligibility. Study eligibility criteria included: 1) age 20-45 years at delivery, 2) a singleton, live birth ≥ 35 weeks' gestation, 3) KP Regional laboratory diagnosis of gestational diabetes by 3-h 100 g OGTT, 4) no personal history of diabetes, or serious medical conditions, 5) complete KPNC medical and delivery records, 6) no plans for an additional pregnancy in the next two years, 7) not taking any medications that alter blood glucose test results after delivery, 8) primary language is English or Spanish, and 9) prenatal infant feeding intentions.

SWIFT Design

A total of 1033 women were included in the SWIFT study. Each participant provided written informed consent for all study procedures, including three in-person study exams, including repeat 2-hour, 75-gram OGTTs, the use of research data for future studies, and collection of clinical and laboratory data (i.e., vital signs, clinical laboratory tests, medical diagnoses, medication use) from the KPNC electronic health records. The three in-person study visits include Baseline Visit 1 (6-9 weeks postpartum), Visit 2 (1 year postpartum) and Visit 2 (2 years postpartum). The primary outcome for the SWIFT is new-onset diabetes after baseline. Diabetes status was monitored after Visit 2 via systematic and annual surveillance of KPNC electronic health record data. Overall retention of the cohort was excellent with 99% having retested at least once for glucose tolerance after baseline. The follow-up study is currently ongoing.

© 2025 Van JAD et al. *JAMA Network Open*.
