# Supplementary material for: Early Postpartum Glucose Tolerance Reclassification by Gestational Diabetes Subtype
Source: JAMA Netw Open. 2025 Nov 10;8(11):e2542668. doi: 10.1001/jamanetworkopen.2025.42668 (PMC12603860; doi:10.1001/jamanetworkopen.2025.42668)
Supplement: Supplement 2. — Data Sharing Statement [file jamanetwopen-e2542668-s002.pdf]

Data Sharing Statement

Van. Early Postpartum Glucose Tolerance Reclassification by Gestational Diabetes Subtype. *JAMA Netw Open*. Published November 10, 2025. doi:10.1001/jamanetworkopen.2025.42668

Data
Data available: No

Additional Information
Explanation for why data not available: This study utilizes data collected from Kaiser Permanente health plan members diagnosed with gestational diabetes who provided written informed consent to participate in the Study of Women, Infant Feeding and Type 2 Diabetes after GDM pregnancy (SWIFT) in 2008-2011. All study definitions, tools, coding manuals, and SAS programs developed in the course of this study will be made available to interested researchers upon request in accordance with the NIH Data Sharing Policy (http://grants.nih.gov/grants/policy/data_sharing) with no charge and with minimal restrictions.

The SWIFT study is a prospective research cohort funded by the NIH that continues in ongoing follow up. After the completion of the SWIFT study, we are also committed to working with the NIH, other NIH grant recipients and the research community in general to make the data more broadly available under data-sharing agreements with other investigators that provide for: (a) a commitment to using the data only for research purposes and not to identify any individual participant; (b) a commitment to access to the datasets on the secure servers behind the Kaiser Permanente Northern California (KPNC) firewall; and (c) a commitment that the data will not be copied or transferred outside of KPNC.

The SWIFT research visit data and the KPNC electronic health records data from study participants used for this analysis is owned by the Kaiser Foundation Health Plan, Inc., Kaiser Foundation Hospitals, Inc., and The Permanente Medical Group, Inc.; it is not owned by the Kaiser Foundation Research Institute. Because of their third-party rights, it is not possible to make the data publicly available without restriction.

In accordance with Division of Research (DOR) and KPNC's mission to conduct research to serve the public good, we will establish collaborative agreements with other investigators who may wish to use data from the SWIFT study for future secondary analyses addressing important research questions. Such collaborative relationships will take into account resource constraints, opportunity costs, and the need to obtain Institutional Review Board approval.
